# Characteristics and impact of environmental shaking in the Taipei metropolitan area

**DOI:** 10.1038/s41598-021-04528-6

**Published:** 2022-01-14

**Authors:** Kate Huihsuan Chen, Ting-Chen Yeh, Yaochieh Chen, Christopher W. Johnson, Cheng-Horng Lin, Ya-Chuan Lai, Min-Hung Shih, Philippe Guéguen, Win-Gee Huang, Bor-Shouh Huang, Kou-Cheng Chen, Chin-Jen Lin, Chin-Shang Ku

**Affiliations:** 1 Department of Earth Sciences, National Taiwan Normal University, Taipei, Taiwan; 2 Los Alamos National Laboratory, New Mexico, USA; 3 Institute of Earth Sciences, Academia Sinica, Taipei, Taiwan; 4 National Center for Research On Earthquake Engineering, National Applied Research Laboratories, Taipei, Taiwan; 5 ISTerre, Université Grenoble Alpes, 38058 Grenoble, France

**Keywords:** Seismology, Environmental impact

## Abstract

Examining continuous seismic data recorded by a dense broadband seismic network throughout Taipei shows for the first time, the nature of seismic noise in this highly populated metropolitan area. Using 140 broadband stations in a 50 km × 69 km area, three different recurring, strong noise signals characterized by dominant frequencies of 2–20 Hz, 0.25–1 Hz, and < 0.2 Hz are explored. At frequencies of 2–20 Hz, the seismic noise exhibits daily and weekly variations, and a quiescence during the Chinese New Year holidays. The largest amplitude occurred at a station located only 400 m from a traffic-roundabout, one of the busiest intersections in Taipei, suggesting a possible correlation between large amplitude and traffic flow. The median daily amplitude for the < 0.2 Hz and 0.2–1.0 Hz frequency bands is mostly synchronized with high similarity between stations, indicating that the sources are persistent oceanic or atmospheric perturbations across a large area. The daily amplitude for the > 2 Hz band, however, is low, indicating a local source that changes on shorter length scales. Human activities responsible for the 2–40 Hz energy in the city, we discovered, are able to produce amplitudes approximately 2 to 1500 times larger than natural sources. Using the building array deployed in TAIPEI 101, the tallest building in Taiwan, we found the small but repetitive ground vibration induced by traffic has considerable effect on the vibration behavior of the high-rise building. This finding urges further investigation not only on the dynamic and continuous interaction between vehicles, roads, and buildings, but also the role of soft sediment on such interaction.

## Introduction

Mounting evidence indicates that seismic noise in cities could be strong enough to contaminate the seismic catalogues used for the detection of earthquakes and tremors [e.g.,^1–5^]. A better understanding of noise produced by both natural and anthropogenic activities is thus crucial to improve the detection of small tectonic events in populated areas. Especially in urban areas with low seismic activity, ambient noise has become a key signal in modern seismology that utilizes seismic surveys for investigating underground structures and seismic microzonation [e.g.,^6–9^]. Ambient noise in cities is driven by numerous human processes including vehicle traffic, metros, airports, and industrial activity [e.g.,^10–13^]. During the COVID-19 outbreak, a strong correlation between the magnitude of seismic noise and human mobility implies that seismic noise in dense urban environments provides a real-time estimate of population dynamics^14–17^. A complete understanding of the spatial and temporal variations of urban induced ground motions is thus necessary for the successful utilization of seismic noise.

Seismic noises generated by cultural or natural origins have different frequency content and spatiotemporal characteristics^18–22^. They can be classified into two types: (1) microtremors (higher than 1 Hz), signals that are mostly induced by human activity and that systematically exhibit daily and weekly variations^12,13,23–25^, or (2) microseisms (below 1 Hz), signals mainly excited by oceanic gravity waves, which can be categorized into primary (0.02–0.1 Hz) and secondary (0.1–0.5 Hz) microseisms^19,22,26–28^. With high levels of background noise found in urban areas, urban seismology has become an active research field due to the increasing number of seismic arrays located in cities [e.g.,^7,10,29^].

**Figure 1:**
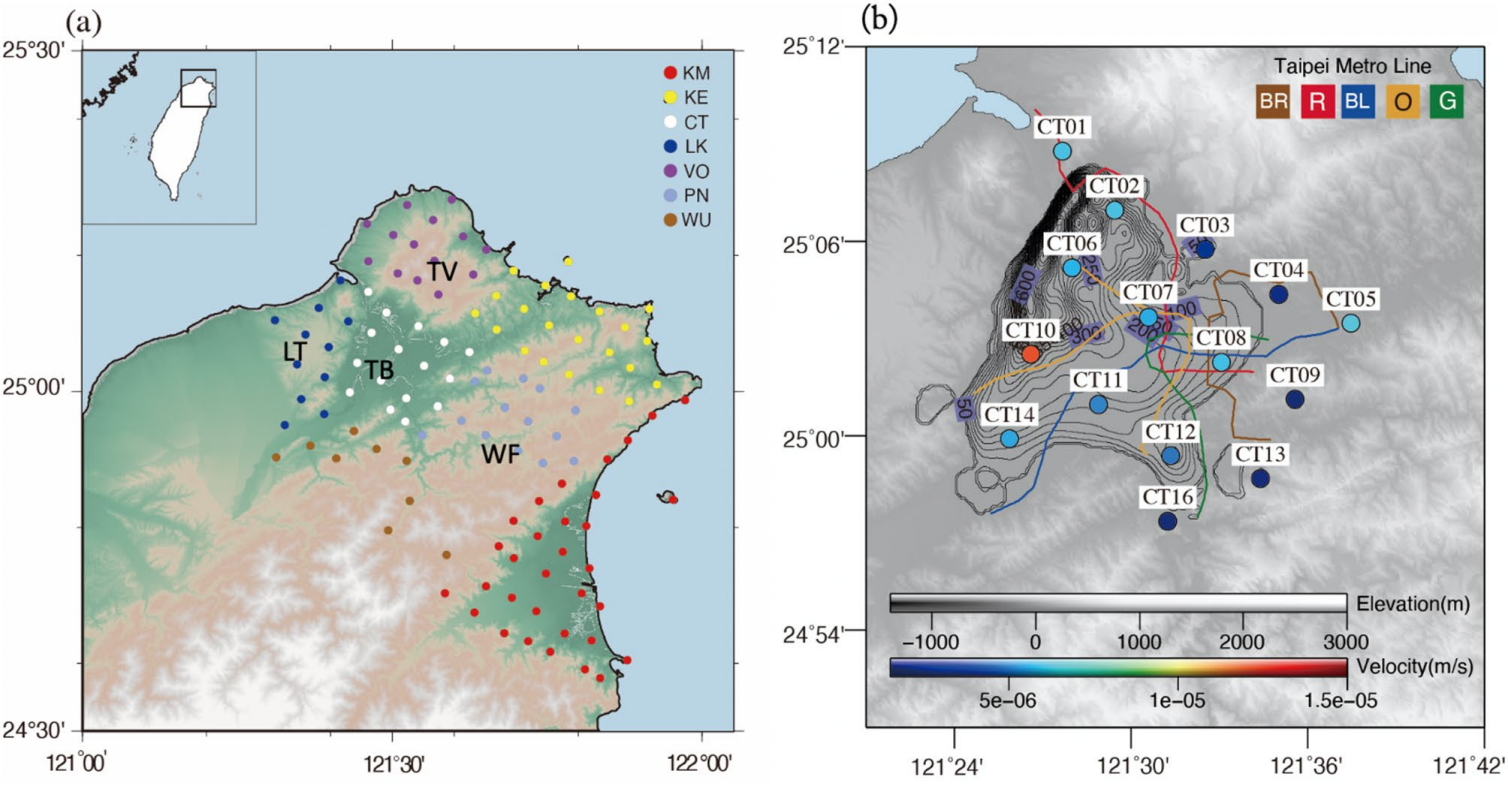
(**a**) Map of northern Taiwan showing the 140 broadband stations in the Formosa Array (FA) located in the Taipei metropolitan area. The stations are divided into seven subareas shown with the different colors and abbreviation for each. The inset map shows the location in Taiwan. WF: the Western Foothills; TV: the Tatun volcanic area; LT: the Linkou Tableland; TB: the Taipei Basin. (**b**) The fifteen CT stations located in the main city of Taipei, with each station color-coded according to the median value of the daily amplitude of the background seismic noise. The colored lines show the Taipei metro system route map with the corresponding color indicating the metro line abbreviation. The contour denotes the Tertiary basement depth contours of the Taipei basin by^52^. (**a**) and (**b**) are generated using GMT − 5.4.4.

Taipei City is the political, economic, and cultural center of Taiwan and hosts a population of 2.7 million people. This metropolitan area is located in a Quaternary sediment-filled basin with low seismicity and composed of four geomorphological elements: the Western Foothills (WF), the Tatun volcanic area (TV), the Linkou Tableland (LT), and the Taipei Basin (TB) (Fig. 1c). The Tectonics and geology around the Taipei metropolitan area can be seen in Supplementary Information Text S1. The Formosa Array (FA), a dense broadband seismic array deployed in 2017 with 140 stations at approximately 5 km spacing, is used to investigate the geometry of the magma chamber beneath the Tatun volcanic area^30–32^. The FA also provides an excellent opportunity to study the characteristics and origin of urban seismic noise that is driven by numerous anthropogenic processes. This study is aimed at exploring the nature of ground vibration rhythm in Taipei metropolitan area to investigate what are the spatiotemporal characteristics and the possible origins of ambient noise in this highly populated city.

## Spectral characteristics of seismic noise in Taipei City

**Figure 2:**
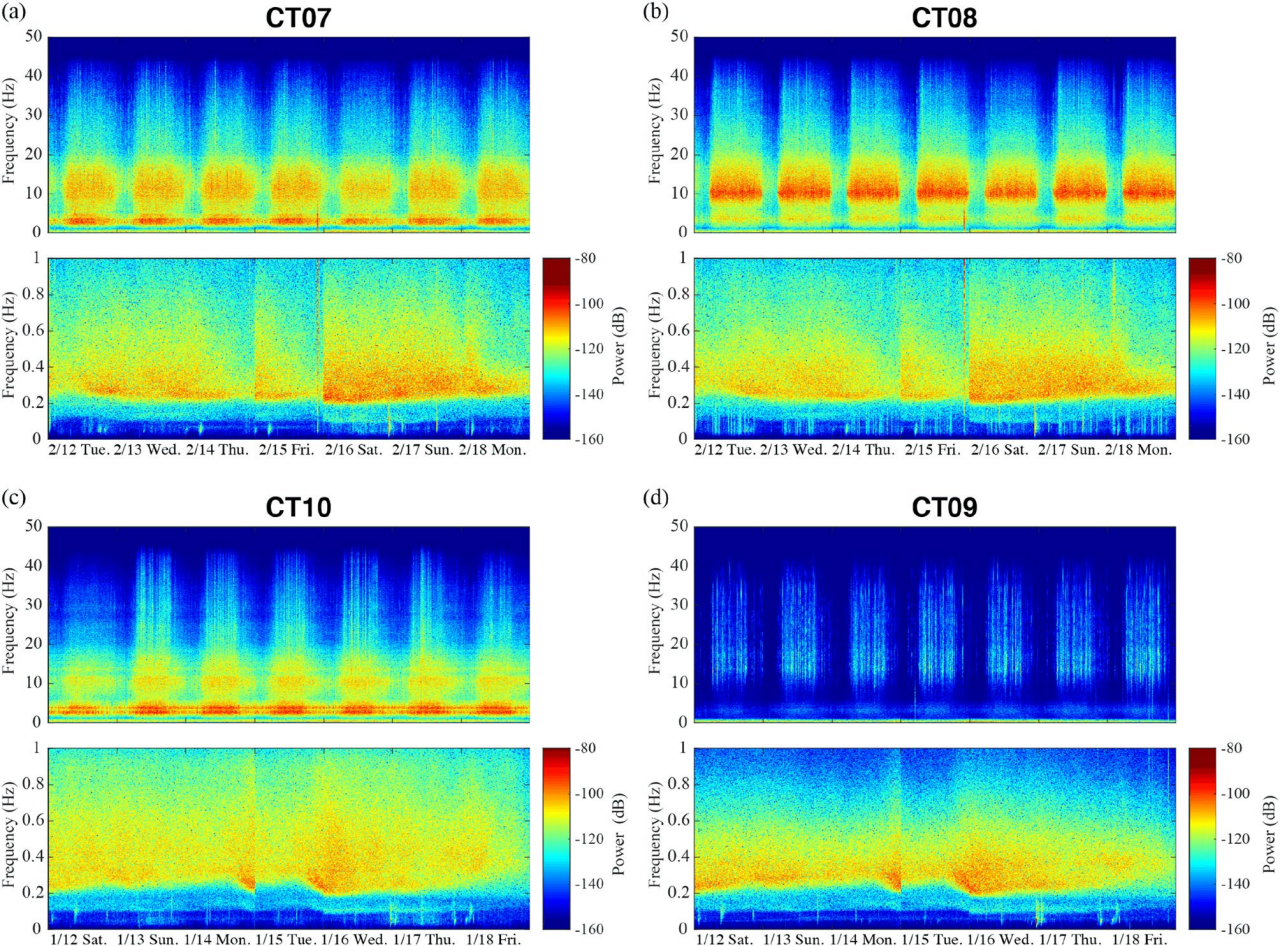
Spectrograms of the vertical seismic data for four stations from the CT subarea. Data for Station (**a**) CT07 and (**b**) CT08 are for the period of February 12 to 18, 2019, and (**c**) and (**d**) for the period of January 12 to 18 in the same year. In each figure, the upper panel shows the frequency range from 0–50 Hz and the lower panel only shows low frequencies, below 1 Hz.

A dense broadband seismic network with a 100 Hz sampling rate was deployed in the Taipei metropolitan area from 2017 to present by the Institute of Earth Sciences of the Academia Sinica and the Taiwan Volcano Observatory at Tatun. The 140 seismic stations were divided into seven subareas (location shown in Fig. 1a) and the fifteen stations in the CT (abbreviation of “city”) subarea located in the highly populated area near the Taipei Metro system (Fig. 1b). We used the vertical component of the continuous seismograms for 2 months from January 1 to February 28, 2019 to explore the characteristics of ambient noise. The data were preprocessed by removing the mean and trend, and deconvolving the instrument response. When calculating spectrograms for a two-month time-series using a 1-h window with a 0.5-h overlap, we found a clear distinction in amplitude between day and night that occurs mainly in the range of 2–20 Hz, as shown by the examples in Fig. 2. Station CT07 is located within 200 m of an underground subway station and main road, and CT08 and CT10 are located adjacent to a main road; all show the strongest spectral energy at approximately 2 and 10 Hz. In contrast, station CT09 located 1270 m from the main road in a relatively remote area where only two buses operate each day, has a weaker amplitude between 2–20 Hz. A closer inspection of the CT09 daily time-series filtered between 2–40 Hz shows impulsive signals occurring between 6:40 am and 6:10 pm that coincide with the start and the end of the bus schedule in this area (Figure S1 in the supplementary file).

In the frequency band below 1 Hz (lower panels in Fig. 2a-d), the highest energy is observed from 0.2 to 0.6 Hz and characterized by different recurring patterns when comparing with the 2–20 Hz frequency band. The 0.2–0.6 Hz frequency band shows longer recurrence times that range from 1.5 to 5 days. At frequencies < 0.2 Hz, short-lived, frequently recurrent energy bursts are observed in the spectrograms. The energy bursts during the same period (from February 12 to 18 in Fig. 2a,b or January 12 to 18 in Fig. 2c,d) show similar arrivals for relatively higher-power spikes at different stations. In visual inspection, most of the high amplitude energy bursts (> − 90 dB/Hz) coincide with local and regional earthquakes (location please see Figure S2), as denoted by grey and blue arrows in the month-long spectrograms shown in Figure S3. The energy burst that persists relatively longer (> one hour) is found to correspond to a teleseismic event as denoted by orange arrow (the corresponding spectrogram can be seen in Figure S4).

**Figure 3:**
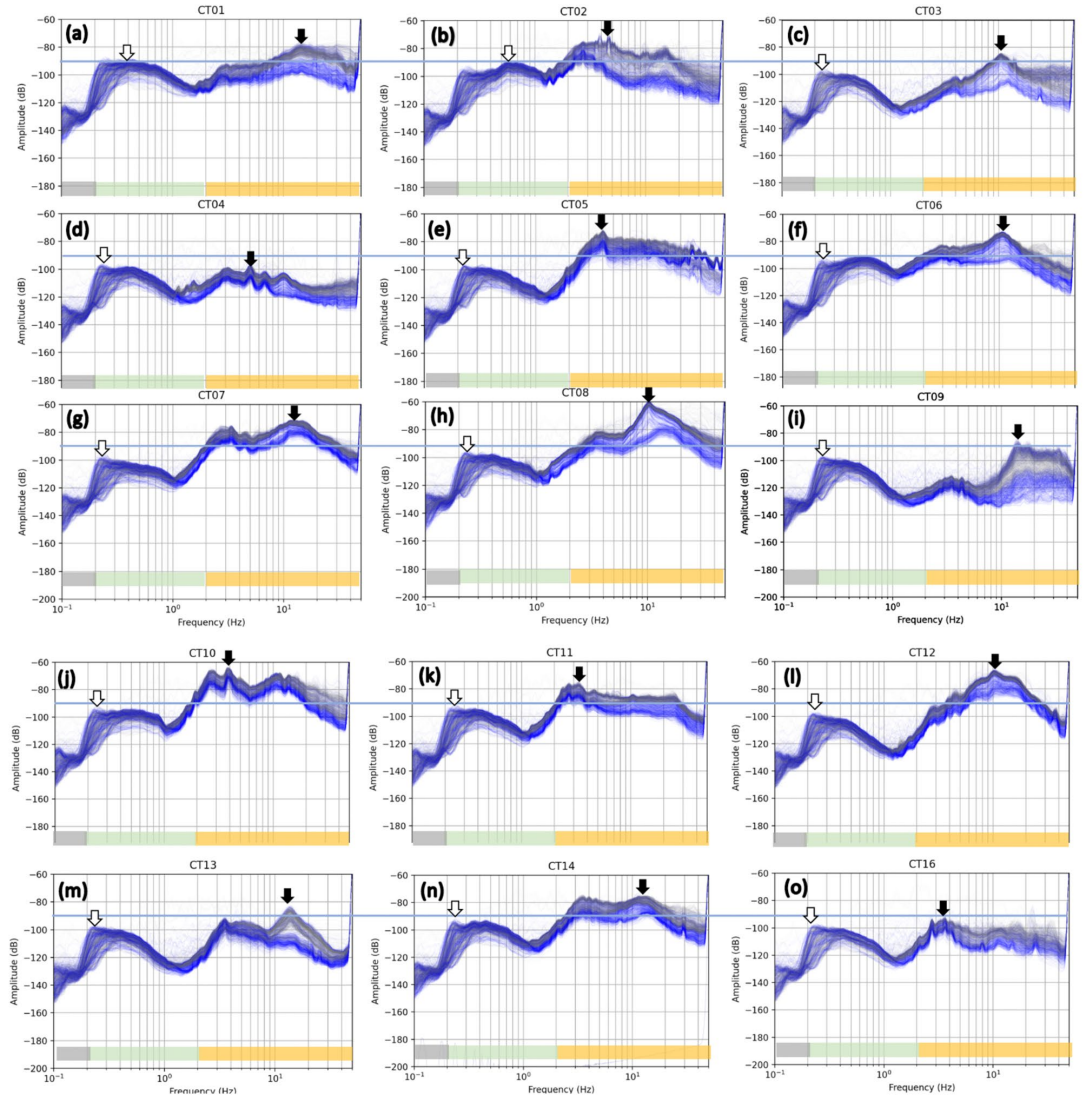
PSD at different stations in Taipei city. (**a**–**o**) show the maps of PSD at stations CT01 to CT16. The hourly vertical seismogram from January to February of 2019 is processed for the plots. Grey and blue lines indicate the data from day (6 am to 6 pm) and night (6 pm to 6 am), respectively. The horizonal bar denotes three different frequency bands of > 2 Hz, 0.2–1 Hz, < 0.2 Hz. Black and white arrows indicate the largest amplitude in > 2 Hz and 0.2–1 Hz bands, respectively. Each curve represents hourly data during the two-month study period. Blue horizontal line denotes − 90 dB for a reference.

The frequency characteristics of the seismic noise can be further studied through a spectral analysis on the continuous data. Using Welch’s technique^33^, the power spectral density (PSD) is first obtained for each station in Taipei city using the first two months data of the study period. As shown in Fig. 3, the three separate frequency bands (> 2 Hz, 0.2–1 Hz, and < 0.2 Hz) are distinct, as denoted by horizontal bars in the bottom. The largest amplitude appears in the band of 2- 20 Hz and smallest in < 0.2 Hz. Note that the PSD distributions for > 2 Hz vary significantly between stations, while they remain highly similar for the 0.2–1 Hz and < 0.2 Hz bands. As revealed by the black arrows in Fig. 3, the highest peak occurs around 10 Hz (at CT01, CT03, CT06, CT07, CT08, CT09, CT12, CT13, and CT14) or 4–5 Hz (at CT02, CT04, CT05, CT10, CT11, and CT16). Among city stations, the smallest amplitudes are observed at CT09, CT13, and CT16 that are located near Western Foothills. These 3 quiet stations also happen to be the farthest from metro lines (> 1.5 km), implying that they are relatively remote from the core population of Taipei city. Note that the temporal behavior in the 2–40 Hz bands reveals a stronger variation when comparing with < 1 Hz frequency (difference between grey and blue lines in Fig. 3). The difference of spectral amplitude between day and night is largest at CT09 for 12–40 Hz, emphasizing the strong temporal change in microtremors is likely a result of extremely low nighttime noise.

CT08 is found to be characterized by the largest amplitude (Fig. 3f), where a traffic-roundabout (i.e., one of the busiest intersections in Taipei) is located within a distance of 400 m. At this station, the waveforms during the rush hour show a repetition of peak amplitude approximately every 3 min (Figure S5), coinciding with the traffic signal cycle lengths for the intersections in Taipei city (92 s stop and 92 s motion). Such correlation confirms the role of vehicle traffic in large ground vibration at > 2 Hz band and motivates further question about whether the traffic-induced vibration has considerable effect on the buildings.

## Traffic induced vibrations in TAIPEI 101?

Numerical studies of building vibration induced by traffic have been undertaken in the cities around the world [e.g.,^34–37^]. How much traffic-induced vibrations impact the structures in the cities has not been fully explored due to their low amplitude comparing with earthquakes. It is worth noting that the largest amplitude in > 2 Hz occurred at station CT08 (Fig. 3h) that is only 1 km from the TAIPEI 101 skyscraper, the 10th tallest building in the world. Is it possible for the traffic induced ground vibration to have an impact on a high-rise building? The building array at TAIPEI 101 is operated by the Institute of Earth Sciences, Academia Sinica, and has routinely recorded building vibrations. This provides a unique opportunity to demonstrate the effects of traffic-induced vibration on the high-rise building.

**Figure 4:**
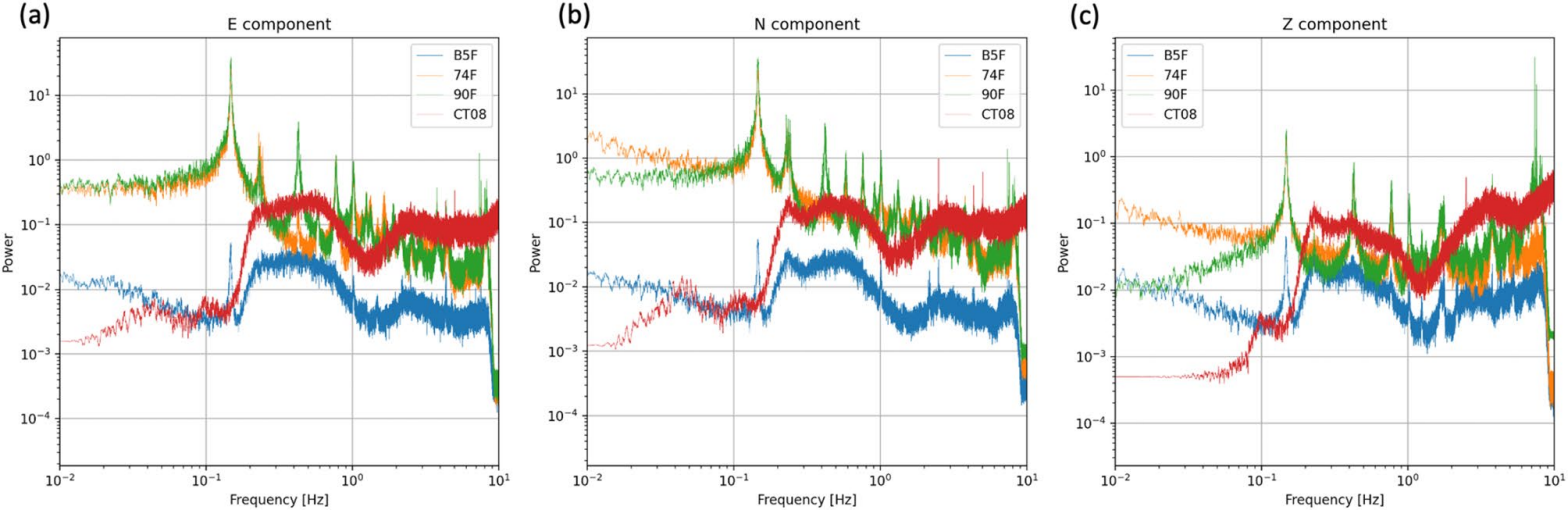
Spectral amplitudes of the four hours data (00:00–04:00) recorded at CT08 and at 5th floor below the ground (B5F), 74th floor (74F), and 90th floor (90F) in TAIPEI 101 on December 5, 2019 for the (**a**) E-W component (**b**) N-S component and (**c**) and vertical component. Different colors represent the spectra at different stations.

The broadband seismometers in TAIPEI 101 were deployed on the 90th (90F) and 75th (75F) floors and below ground on the B5th (B5F) floor, to continuously record the vibration at 20 sample per second. To avoid aliasing, the frequency is confined within 10 Hz for further comparison with CT08. Figure 4 shows the acceleration spectra in three components at the station CT08 and the building array during the quiet nighttime hours from 00:00 to 04:00 AM on December 5, 2019. Note that the spectral behavior of B5F is almost identical with CT08 above 0.1 Hz, showing energy concentration at 0.2–1 Hz and > 2 Hz at CT08 with ~ 10 times smaller amplitude. The vertical component reveals relatively higher amplitude for both CT08 and B5F at > 2 Hz band and more pronounced modes of the building at higher frequencies as evident by spectral peaks that correspond to the higher floors. Such similar spectral behavior implies the origin of ground and building vibration at higher than 0.1 Hz is likely the same. The spectral amplitude at 74F and 90F, on the other hand, reveals multiple peaks as different oscillation modes of TAIPEI 101. The largest peak occurs at ~ 0.15 Hz, corresponding to the first-mode of TAIPEI 101. The overall amplitudes for higher floors are similar with CT08 but greater than B5F, over a wide range of frequency from 0.1 to 10 Hz.

**Figure 5:**
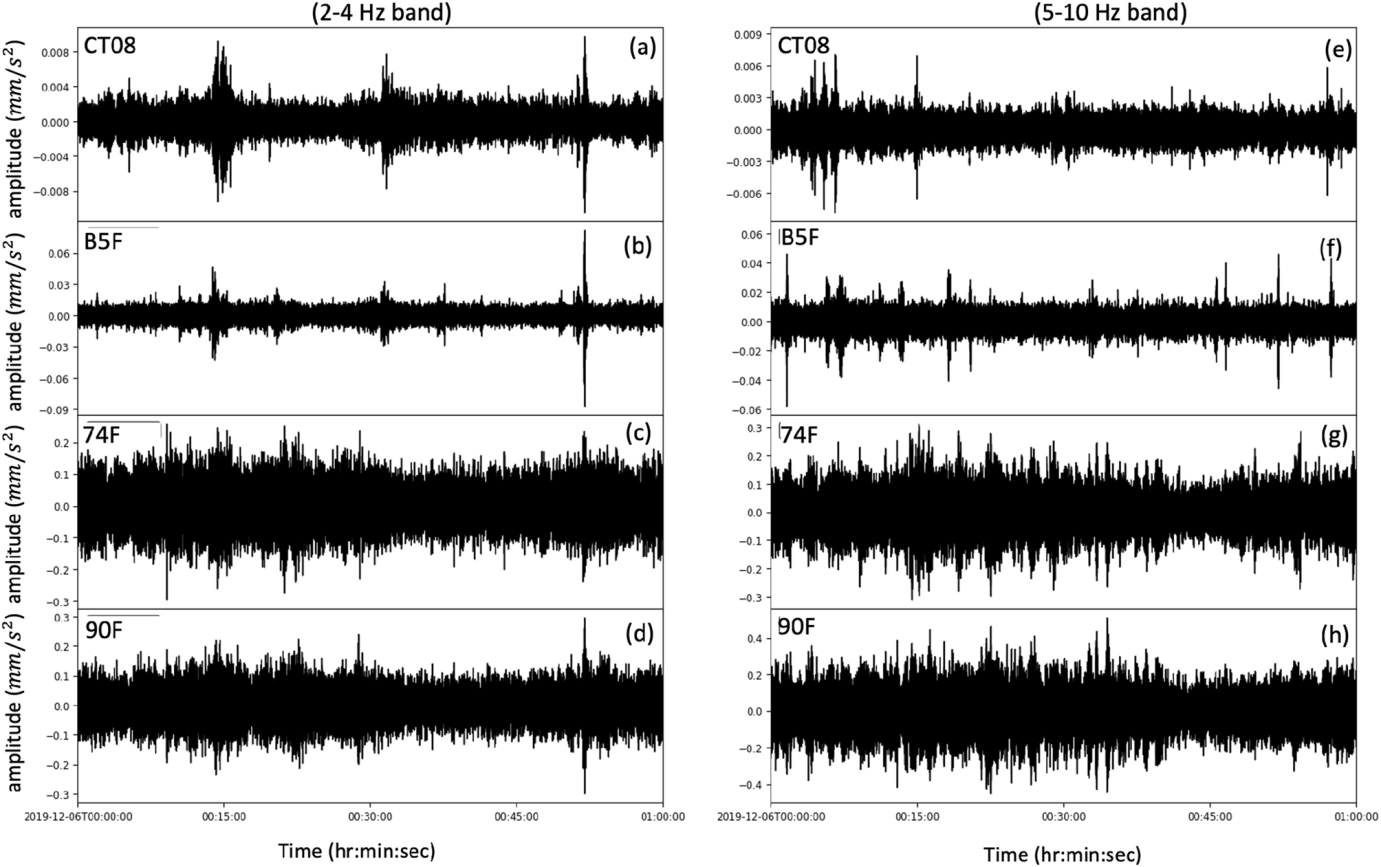
The one-hour signals (00:00–01:00 AM) recorded at CT08 and by the building array at B5F, 74F, and 90F for (**a**-**d**) 2–4 Hz band and (**e**–**h**) 5–10 Hz band on December 5, 2019. The selection of bandpass filters of 2–4 Hz and 5–10 Hz follows the energy concentration in the spectrograms shown in Fig. 2b while the cutoff below 10 Hz instead of 20 Hz is due to the Nyquist frequency of building array.

We found that the ground vibration recorded at CT08 reveal clear synchronization at the B5F for 2–4 Hz in the midnight (00:00 to 01:00), as shown in Fig. 5a,b. At the 74F and 90F (Fig. 5c,d), the temporal fluctuation in peak amplitude is less similar with CT08 except for the highest peak at 00:52:00. At higher frequency of 5–10 Hz band, however, the synchronization between ground and building stations is no longer obvious in the visual inspection of Fig. 5e-h. Given that the fundamental frequency of the soft sediment underneath CT08 and TAIEPI 101 is about 1–2 Hz (Lin et al., 2014), it is likely for the lower frequency energy of the traffic induced signals (2–4 Hz) being preserved easily compared with 5–10 Hz energy.

**Figure 6:**
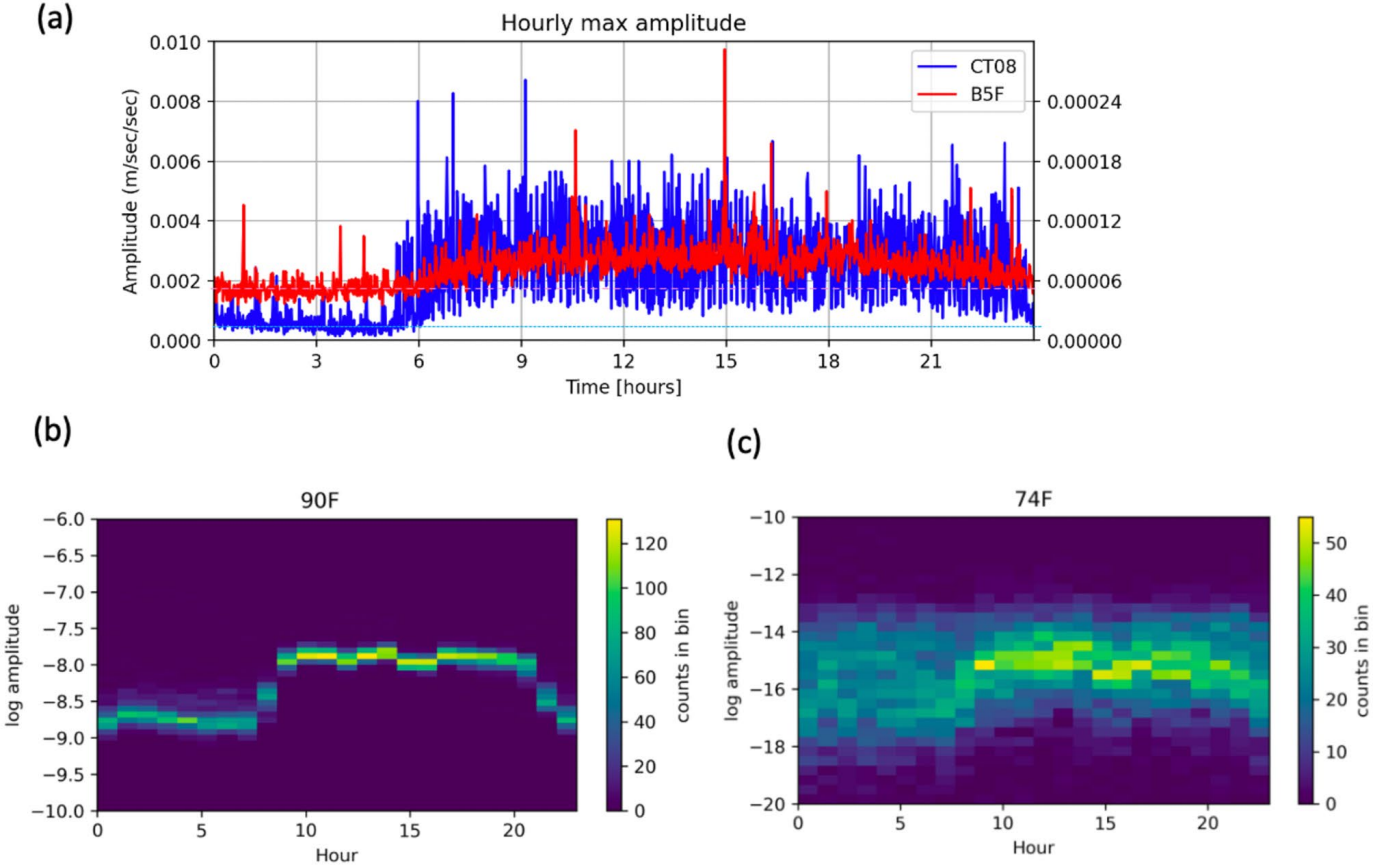
(**a**) The variation of peak amplitude observed on December 5, 2019 for CT08 in Formosa Array and B5F in TAIPEI 101. Horizontal blue and pink dashed lines denote the averaged amplitude determined using quiet period from 0 to 4 AM for CT08 and B5F of TAIPEI 101, respectively. (**b**) Density plot for annual average of hourly amplitude at 90F of TAIPEI 101 using 365 days continuous data in 2019. The color represents data concentration of fundamental mode. (**c**) Density plot for annual average of hourly amplitude at 74F of TAIPEI 101 using 365 days continuous data.

When the focused period is increased from one hour to 24 h, the hourly variation in peak amplitude for CT08 and B5F can be shown in blue and red lines in Fig. 6a, respectively. In spite of different amplitude, we found that both of the stations reveal the similar trend of (1) the arrival of elevated amplitude starting at 6 AM (2) the end of the elevated amplitude taking place at 11 PM. To further understand whether such hourly variation can be seen in high floors, the power spectral density for fundamental frequency of TAIPEI 101 at ~ 0.15 Hz (the largest peak in spectra shown in Fig. 4) was obtained using one-year continuous data in 2019 for statistically meaningful representation. When the temporal fluctuation in the first mode of natural frequencies is represented by the density plots in Fig. 6b,c, the energy concentration appears to be more significant at 90F comparing with that at 74F, while the general pattern remains the same. That is, the amplitude remained stably high from 9 AM to 8 PM at 74F and 90F. Such long-lasted high amplitudes of first-mode motion of TAIPEI 101 exhibit very similar temporal pattern with the peak amplitude recorded at B5F and the CT08 station 1 km away. This suggests the common origin(s) of the temporal variation in (1) peak amplitude recorded at CT08 and the B5F and (2) the first-mode vibration observed at 74F and 90F.

In fact, the continuous vibration of TAIPEI 101 has been explored to correlate with wind speed and temperature, and thus reveal strong seasonality^38^. We argue that the short-term behavior of a high-rise building (i.e., hourly) may have different origin. Given that the wind perturbation does not usually concentrate in a certain hour of the day and that the temperature change has abrupt decay before and after noon, the observed first-mode vibration centroided near 0.15 Hz may be explained by the natural sources. The dynamic interaction between the visitors and the vibration of TAIPEI 101 could be also excluded due to the business hours is from 11:00 to 21:30. Although the natural frequencies of vehicles (> 2 Hz) are generally higher than the fundamental frequencies of the high-rise building and very small in amplitude comparing with earthquakes, the traffic induced vibrations may play a certain role on adjusting the short-term variation in the building vibration. Under which condition the influence is more significant and how much their coupling reflects the near-surface soil condition can be further studied in the future.

## Temporal variation in the median seismic amplitude

**Figure 7:**
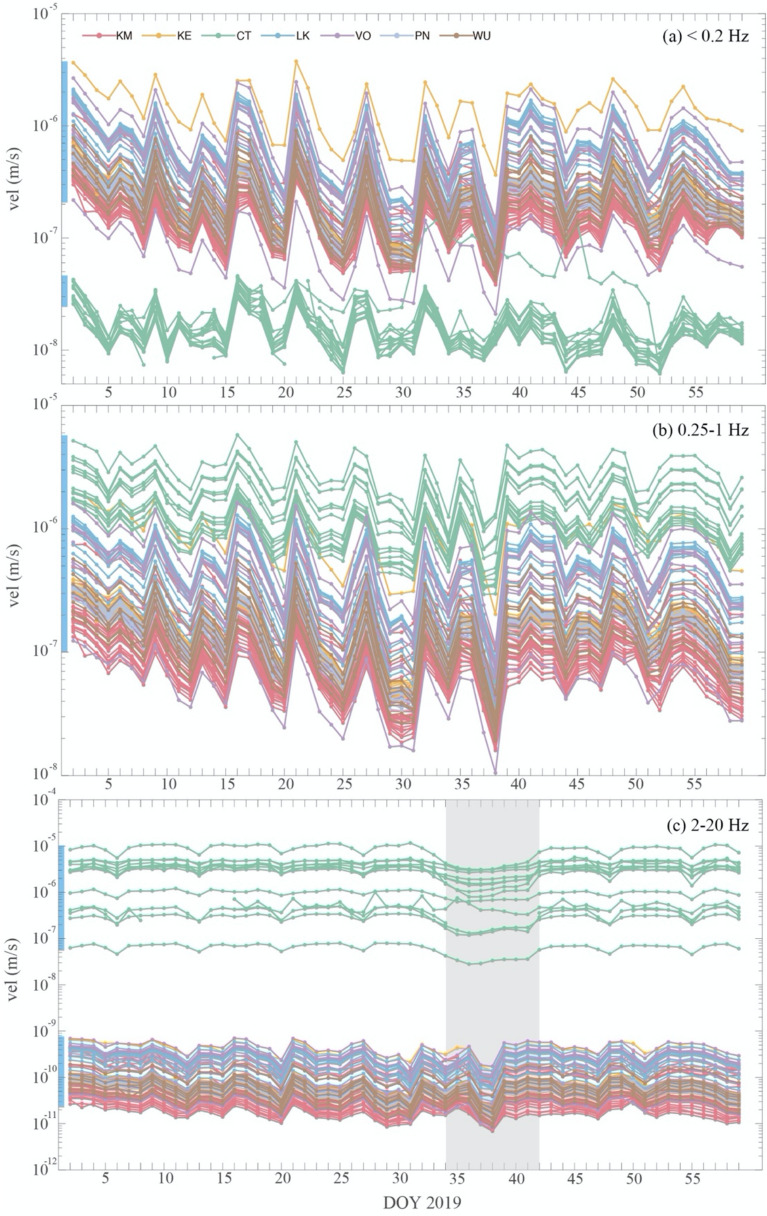
The median values of the daily amplitude at stations in different subareas for three frequency bands: (**a**) < 0.2 Hz, (**b**) 0.25–1 Hz, and (**c**) 2–20 Hz. DOY, day of year. The blue vertical bar on the *y*-axis shows the range of first points in the two-month time-series for the various stations. The grey vertical area in (**c**) indicates the Chinese New Year holiday.

To capture the temporal variation of ambient noise in the city and the wider metropolitan area, the median value of daily amplitude is displayed in Fig. 7. The median daily amplitude at all 140 stations was now calculated and displayed in Fig. 7, to further understand how the temporal patterns in the three frequency bands vary spatially. At frequencies below 0.2 Hz, the temporal variation in six subareas outside Taipei City are consistent and have a small range of amplitude from $2.1\times {10}^{-7}$ to $2.5\times {10}^{-6}$ m/s (denoted by the vertical blue bar on the y-axis in Fig. 7a, which is the range of the initial amplitude in the examined time-series). However, the CT stations in the city show initial amplitudes between $1.3\times {10}^{-8}$ and $3.5\times {10}^{-8}$ m/s, which is approximately an order of magnitude smaller than the other six subareas. For the 0.25–1.0 Hz frequency band, a similar initial amplitude range of $1.1\times {10}^{-7}$ to $4.6\times {10}^{-6}$ m/s is observed, but in this case the largest amplitude is observed at the CT stations (Fig. 7b). We suspect that the effect of basin resonance may play a role, which will be addressed in the next Sect.

Note that in Fig. 7a,b, the temporal variation is consistent for stations in different subareas throughout the range of amplitudes, as the median daily amplitude for the < 0.2 Hz and 0.25–1.0 Hz frequency bands are mostly synchronized. This suggests the sources are a persistent disturbance distributed over a large area. The higher frequency band of 2–20 Hz reveals higher amplitudes at the CT stations with values ranging from 5.5 $\times {10}^{-7}$ to $1.0\times {10}^{-5}$ m/s (blue vertical bar in Fig. 7c). The temporal variation at the CT stations indicates the influence of human activity with the lowest amplitudes observed consistently during the weekends and the Chinese New Year holidays (grey area in Fig. 7c). The median amplitudes for the stations outside the city exhibit a surprisingly similar time pattern, yet with reduced amplitudes, suggesting a common driving force. We found that only the CT stations show significant frequency dependency; the vibration at 2–20 Hz reveals widely different temporal behavior compared with that at 0.25–1 Hz and < 0.2 Hz frequency bands (green curves in Fig. 7).

As shown in Figure S6, the spectrograms from sample stations in each subarea confirm that the higher frequency band is consistent with energy concentrating above 2 Hz, yet is weaker than observed in the CT subarea and sometimes lacks a weekly variation (Figure S6d-e). At the lower frequency band below 1 Hz, there is a clear boundary at 0.25 Hz for all stations outside the city (Figure S6), whereas in the city (CT stations) the boundary is at 0.2 Hz (Fig. 2). Note that at the stations outside the city, the amplitude of the lower (< 1 Hz) frequency band is commonly found to be much stronger than the higher frequency bands (> 2 Hz). These long period signals are very different from what is observed in the CT stations, where the anthropogenic noise at 2–20 Hz remains strongest near the main roads and metro rail lines.

### Seismic site effects due to thick basin sediments

The amplitude of land-recorded, secondary microseismic noise is found to be controlled by the sediment thickness below the source region and the source location relative to the local ocean depth^39,40^. At the top of the Taipei Basin, there is an approximately 120-m thick Quaternary sedimentary layer that is characterized by a P-wave velocity of 0.45–2.2 km/s and an S-wave velocity of 0.17 to 0.88 km/s^41^. This Sungshan Formation has been recognized to be responsible for the amplification of seismic waves during earthquakes [e.g.,^41,42^]. The depth distribution of the Shungshan formation is shown by Fig. 1b, showing a deepest part located to the NW of the basin. The anomalous thickness of the soft sediment in the basin (CT subarea) leads to a dominant frequency of 0.3–1.4 Hz [e.g.,^43,44^], which provides an alternative interpretation for the large amplitude of seismic waves in the 0.25–1 Hz frequency band (green lines in Fig. 7b). Based on the microtremor H/V measurements, the fundamental frequency in the Taipei Basin is approximately 0.5 Hz, which is lower than those in sites on the plain and hill that are characterized by frequencies higher than 1 Hz^45^. In fact, the relatively high amplitude in the 0.25–1 Hz band is found at CT01 (− 90 dB), CT02 (− 90 dB), CT06 (− 93 dB), CT14 (− 95 dB), and CT10 (− 95 dB) in the PSD (Fig. 3). These stations are located at the sites with thicker Sungshan Formation, as shown by the contour in Fig. 1b. The only exception occurs at CT10 near the river where the soft soil is thin but is close to the ocean and thus, the influence of oceanic forcing is large comparing with the rest of the city stations. We argue that the higher amplitude at 0.25–1 Hz events at CT stations is likely a result of the resonance frequency of the Sungshan Formation.

### The regional dependence of the seismic amplitude during typhoons

**Figure 8:**
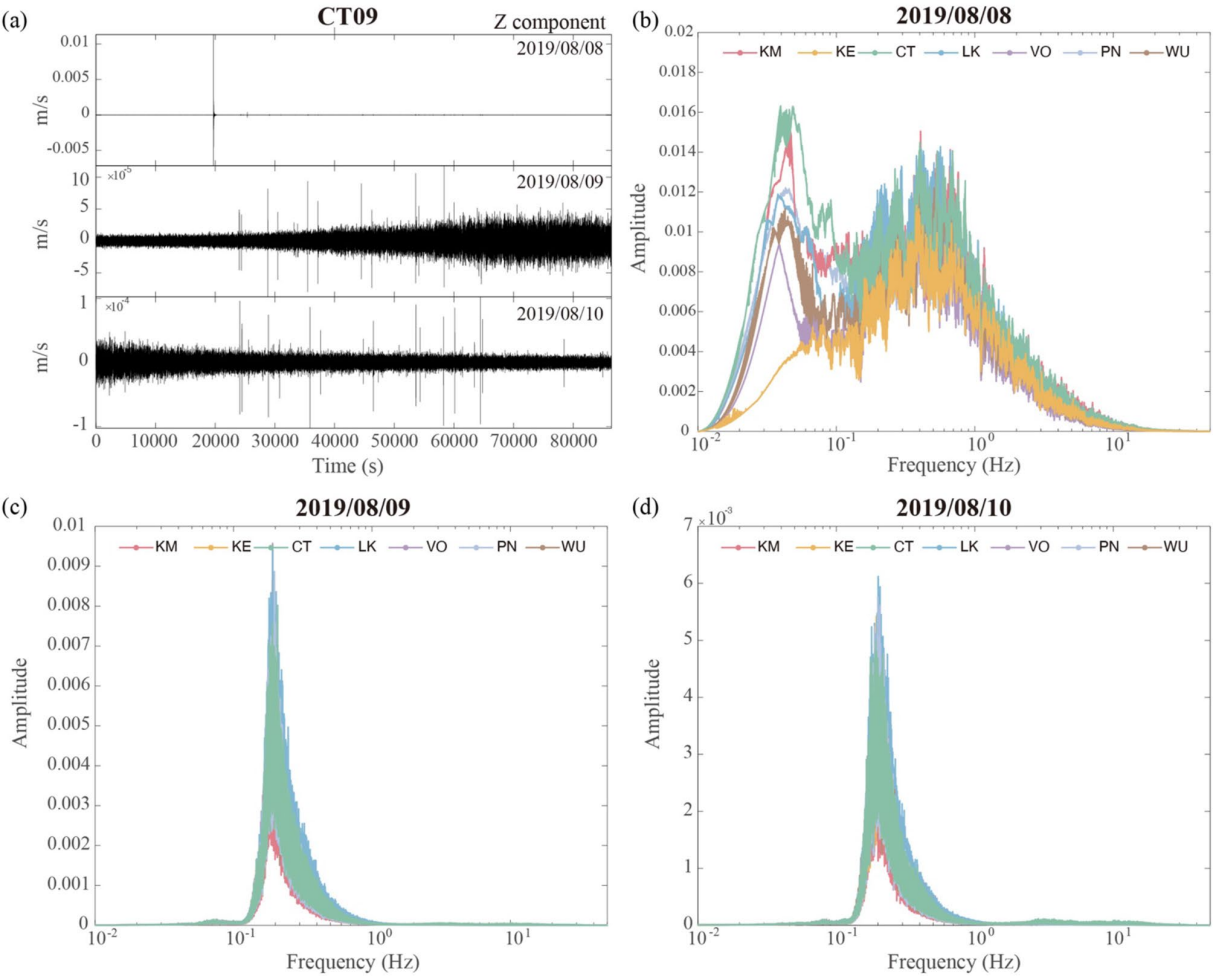
(**a**) The waveforms recorded at Station CT09 on August 8 to 10 (upper to bottom panels). The peak indicates a M6.2 earthquake near Yilan. (**b**-**d**) The median spectra from different subareas of the FA on August 8, 9, and 10.

It is also possible that the strong wind restricted to the surface has impact on the city center due to the shaking of the buildings^46^. To understand the seismic response due to wind disturbance, the waveform, spectrum, and hourly median amplitude during a strong typhoon, the Likima typhoon that occurred from August 7–9, is now examined. As shown in Fig. 8, the typhoon land warning was issued at 5:30 pm on August 7, whereas early in the morning there was a M6.2 earthquake offshore of the northeast of Taiwan near Yilan County (sharp peak in upper panel of Fig. 8a). A maximum wind velocity of 21.1 m/s was recorded at the weather station in Taipei City at 7:00 pm on August 9. The corresponding spectral behavior reveals a peak near 0.2 Hz and a frequency range of 0.1 to 1 Hz (Figs. 8c,d). On August 8, the spectra present a more complicated pattern with two peaks at 0.03–0.06 Hz and 0.2–1 Hz, the largest amplitude being recorded at the CT stations (Fig. 8b). Given that the typhoon did not arrive until the evening of August 8, the spectral behavior for this day may combine the effects from the M6.2 earthquake, anthropogenic noise, and the growing wind field.

The spatial distribution of the maximum amplitude in the Formosa Array is shown in Figs. S6, S7. The maximum hourly-averaged seismic energy is found to occur along the coast of the LK and VO subareas and the port of Keelung, which remained the same at different hours of a day. Comparing the seismic data with precipitation and wind velocity at 9 am and 10 am, it was found that the maximum precipitation is highly variable over time without clear correlation with seismic data (Figures S7c and f). However, the hourly averaged wind speed is persistent with time and is concentrated at two locations on the northern coast (dark red and orange triangles in Figures S7b and e), which is more consistent with the seismic data. Note that the spatial correlation between strong winds, high rainfall intensity, and the largest seismic amplitude in the 0.25–1 Hz is not clear, which could be due to a lack of weather stations in the LK subarea. As the typhoon originally developed offshore of Taiwan to the east, the impact of terrain on typhoon circulation may play an important role in generating the enhanced amplitude along the coastal stations in the VO and LK subareas. The basin area (where CT stations are located) however, was not found to experience the largest seismic amplitude at 0.25–1 Hz during the strong typhoon. We argue that during typhoons, the 0.25–1 Hz seismic waves are mainly generated by strong winds. Without typhoons, the frequency of approximately 0.5 Hz could be amplified due to the presence of thick and soft sediments in the basin. The largest noise amplitude in the 0.25–1 Hz range observed in the CT subarea illustrated in Fig. 7b, is interpreted by the ambient noise induced resonance in deep sedimentary basin.

## Possible origin of the seismic noise

The major source of vibration excitations for a moving train is the rail-wheel-body interaction, and^47^ reported that separate frequency ranges of 0.8–4 Hz and 8–15 Hz are responsible for train-body skipping from pavement unevenness and axle hop movement, respectively^48^ further demonstrated that the main frequency can be switched between 2 and 10 Hz when the geometry of the speed reducer and suspension system changes. Using microelectromechanical system (MEMS) sensors to record the vibration of moving vehicles in Taipei City, we confirmed that a moving metro train, bus, and truck exhibit dominant frequencies of 2–10 Hz. Therefore, the energy concentrations at approximately 2 Hz and 10 Hz are interpreted as city traffic induced vibrations.

Natural sources contribute to the lower frequency band below 1 Hz, and these sometimes overlap with anthropogenic signals^7,49^. Oceanic infragravity waves have long been recognized as the most likely source of seismic free oscillation. Atmospheric disturbances produce a pressure source at the seafloor, and the induced oceanic infragravity waves can excite a very long period seismic hum (frequency < 0.02 Hz). The nonlinear interactions among wind waves are also able to produce ocean infragravity waves^27,50^. Since the behavior of oceanic infragravity waves is depth dependent and exhibits a strong coupling with seabed topography, both oceanic and atmospheric forcing is likely responsible for the observed microseisms in Taipei. In the present observation of temporal variation over the seismic stations, the < 0.2 Hz band demonstrates a stronger amplitude near the shore, indicating that the seismic waves in this frequency band are excited more efficiently. The ocean broadband seismometers (OBS) deployed offshore of Taiwan have detected typhoon-excited microseisms at 0.085–0.2 Hz and 0.15–0.5 Hz^8,51^, suggesting that the driving force for < 1 Hz seismic signals is more closely associated with ocean waves. The possible relationship with air pressure, average wind speed, and wind velocity was also examined. Five weather stations in the Taipei metropolitan area were selected for comparison with the nearest FA station. The weather observations (daily average air pressure, wind velocity, and wind gust for maximum speed) were cross-correlated with the seismic noise daily median amplitude for the three different bands. As shown in Table S1, among the five station sets, the largest cross-correlation coefficient (*ccc*) is found at KM26 and the Suao coastal station, where the *ccc* between seismic noise and wind gusts reaches 0.62 for the < 0.2 Hz frequency band and 0.59 for the 0.25–1 Hz band. The lowest *ccc* values are observed at the Wufenshan and Banqiao weather stations, which are located away from the coast. At the three weather stations on or near the coast, the higher *ccc* can be seen (1) at the lowest frequency band of < 0.2 Hz, and (2) for wind gusts (KM26 versus Suao and KM12 versus Yilan) and wind velocities (LK01 versus Tamsui). The lower *ccc* at the inland stations suggests that the coastal areas may provide an environment that allows for a more direct association between seismic noise and changing wind conditions. Such association, however, is only modest due to the generally lower ccc of < 0.7.

## Conclusion

Continuous data recorded at the 140 stations located in the Taipei metropolitan area provides a unique opportunity to demonstrate the spatiotemporal characteristics of weak ground motion in an urban area. We find three types of seismic noise that are characterized by dominant frequencies of > 2 Hz, 0.25–1 Hz, and < 0.2 Hz. At stations in the city, the energy in the 2–20 Hz frequency band is dominant with a relatively large amplitude. The amplitude is lowest on the weekends and during the Chinese New Year holidays, suggesting a strong association with human activities. In the city, a strong variation appears in amplitude and temporal patterns, indicating that the traffic-induced vibration is localized and the effect varies from station to station.

In the relatively remote areas on the hill and near the coast, the human-induced ground shaking is weaker and the natural sources, such as oceanic and meteorological effects with frequency content < 1 Hz, appear to modulate the observed temporal variation. In the two-month study period from January 1 to February 28, 2019, the daily median amplitude for the < 0.2 Hz and 0.25–1 Hz frequency bands reveal surprisingly consistent patterns at all stations, indicating a common driving force. The largest amplitudes at the beginning of the time-series for 2–10 Hz, 0.25–1 Hz, and < 0.2 Hz were $1.0\times {10}^{-5}$, $2.5\times {10}^{-6}$, and $4.5\times {10}^{-6}$ m/s, respectively. Thus, from maximum amplitude, anthropogenic activities are able to produce weak ground shaking that is 2.2–4.0 times greater than meteorological sources. From all amplitudes from various stations, such ratio can range from 2 to 1500. Naturally occurring noise from oceanic and atmospheric sources primarily contribute to the lower frequency band (< 1 Hz). We found the time-series for the < 0.2 Hz and 0.25–1 Hz seismic data show higher similarity with the recorded wind velocity. Special attention was given to the high amplitudes of 0.1–1 Hz observed at city stations in the Taipei Basin. Given that the resonant frequency of the sedimentary basin (an approximately 120-m thick soft sediment) was centered around 0.5 Hz, it is argued that the regional dependency of amplitude for the 0.25–1 Hz energy is likely controlled by the site effect.

The traffic induced ground vibration for the > 2 Hz band was found to be largest at CT08, where the tallest building in Taiwan (TAIPEI 101) is located just 1 km away. The building array in TAIPEI 101 composed of broadband seismometers on the 90th (90F) and 75th (75F) floors and below ground on the B5th (B5F) floor allows us to demonstrate the effect of traffic-induced vibration on a high-rise building. During a quiet period of a day, the synchronized peaks in waveforms and highly similar spectral behavior can be observed at CT08 and B5F of TAIPEI 101. In addition, the hourly variation of peak ground vibration appears to be similar with the building vibration revealed from peak amplitude at B5F and first-mode variation at the 74F and 90F. We argue that the traffic induced vibration may have impact on the vibration behavior of a high-rise building. The continuous, repetitive ground vibration, although very small comparing with earthquakes, could potentially play a role in adjusting the short-term behavior of the buildings in a densely populated city.

## Supplementary Information

Supplementary Information.
