# Supplementary material for: Characteristics and impact of environmental shaking in the Taipei metropolitan area
Source: Sci Rep. 2022 Jan 14;12:743. doi: 10.1038/s41598-021-04528-6 (PMC8760259; doi:10.1038/s41598-021-04528-6)
Supplement: Supplementary file 1 — Supplementary Information. [file 41598_2021_4528_MOESM1_ESM.docx]

**Supplementary Information**

**Whispering of the city:
Characteristics and origin of environmental shaking in the Taipei metropolitan area**

* Kate Huihsuan Chen^1^([katepili@gmail.com](mailto:katepili@gmail.com))
Ting-Chen Yeh^1^ ([yehtingchen@gmail.com](mailto:yehtingchen@gmail.com))
Yaochieh Chen^1^ ([yaochieh77@gmail.com](mailto:yaochieh77@gmail.com))
Christopher W. Johnson^2^ ([cwj@lanl.gov](mailto:cwj@lanl.gov))
Cheng-Horng Lin^3,4^ ([lin@earth.sinica.edu.tw](mailto:lin@earth.sinica.edu.tw))
Ya-Chuan Lai^4^([yclai@narlabs.org.tw](mailto:yclai@narlabs.org.tw))
Min-Hung Shih^4^([shihmh@narlabs.org.tw](mailto:shihmh@narlabs.org.tw))
Philippe Guéguen^5^ ([philippe.gueguen@univ-grenoble-alpes.fr](mailto:philippe.gueguen@univ-grenoble-alpes.fr))

Win-Gee Huang^3^ (wgee@earth.sinica.edu.tw)

Bor-Shouh Huang^3^ (hwbs@earth.sinica.edu.tw)

Kou-Cheng Chen^3^ ([chenkc@earth.sinica.edu.tw](mailto:chenkc@earth.sinica.edu.tw))

Chin-Jen Lin^3^ (youngman@earth.sinica.edu.tw)

Chin-Shang Ku^3^ ([backnew@earth.sinica.edu.tw](mailto:backnew@earth.sinica.edu.tw))

1.Department of Earth Sciences, National Taiwan Normal University, Taipei, Taiwan

2.Los Alamos National Laboratory, New Mexico, USA

3. Institute of Earth Sciences, Academia Sinica, Taipei, Taiwan

4. National Center for Research on Earthquake Engineering, National Applied Research Laboratories, Taipei, Taiwan

5. ISTerre, Université Grenoble Alpes, Grenoble 38000, France

**Text S1**

**[Tectonics and geology around the Taipei metropolitan area]**

The Taipei metropolitan area is located near the junction between Taiwan orogen and the Ryukyu arc as a result of collision between the Luzon arc and Eurasian plate 10 million years ago (Chen and Teng, 1996; Teng, 1996). During the collision, the sedimentary sequences of the continental margin was developed into the folded and faulted Teritary strata of the Western Foothills (WF in Figure 1a). The subsequent flip of subduction polarity in the late Quaternary (Suppe, 1984) terminated the collision and led to the collapse of the mountain belt, which resulted in the subsidence of the Taipei basin in the frontal part of orogen. The Taipei metropolitan area is composed of four geomorphological elements: the Western Foothills, the Tatun volcanic area, the Linkou Tableland, and the Taipei Basin (Figure 1a). Taipei Basin is filled with Quaternary sediments that consist of interbedded gravel, sand, and mud on the deformed Teritary basement. The basement is deepest (670 m) toward the northwest corner of the basin (Figure 1b). To the north, the Tatun volcanic area (TV) is composed of more than 20 volcanoes and is covered with andesitic to basaltic rocks. To the west of the basin, the Linkou Tableland is capped with lateritics soil with Pliocene-Pleistocene conglomerates, sandstones, and mudstones. The Western Foothills (WF) to the east is composed of clastic sedimentary rocks with ages of Oligocene to Quaternary (Chen and Teng, 1990; Teng et al., 1996).

**Table S1**

Cross-correlation coefficients between the seismic data with different frequency bands and weather observation data.

| FA station vs.  weather station | Frequency range | Air pressure | Averaged wind speed | Wind gust |
| --- | --- | --- | --- | --- |
| KM26 vs. Suao | **< 0.2 Hz** | 0.24 | 0.34 | **0.62** |
|  | 0.25-1 Hz | 0.23 | 0.28 | **0.59** |
|  | 2-20 Hz | 0.15 | 0.15 | 0.45 |
| KM12 vs. Yilan | **< 0.2 Hz** | 0.26 | 0.43 | 0.48 |
|  | 0.25-1 Hz | 0.29 | 0.33 | 0.40 |
|  | 2-20 Hz | 0.41 | 0.32 | 0.33 |
| LK01 vs. Tamsui | **< 0.2 Hz** | 0.34 | 0.49 | 0.39 |
|  | 0.25-1 Hz | 0.34 | 0.47 | 0.39 |
|  | 2-20 Hz | 0.38 | 0.47 | 0.45 |
| KE 11 vs. Wufenshan | **< 0.2 Hz** | 0.31 | 0.21 | 0.23 |
|  | 0.25-1 Hz | 0.34 | 0.15 | 0.13 |
|  | 2-20 Hz | 0.39 | 0.17 | 0.09 |
| CT14 vs. Banqiao | < 0.2 Hz | 0.13 | 0.27 | 0.28 |
|  | **0.25-1 Hz** | 0.38 | 0.33 | 0.44 |
|  | 2-20 Hz | 0.15 | 0.27 | 0.15 |


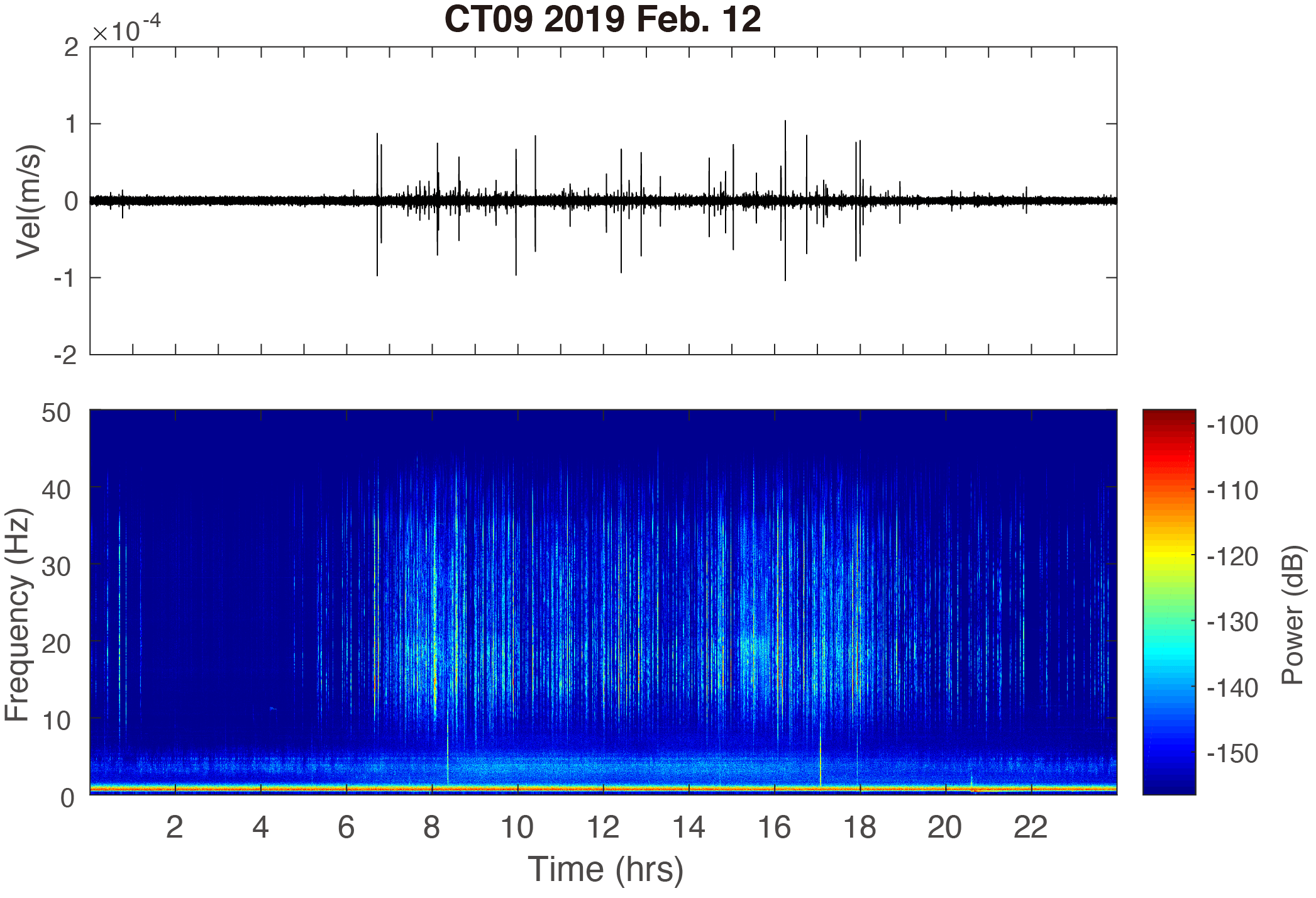


Figure S1. Vertical component of the unfiltered waveform and spectrogram of the seismic data on February 12 at Station CT09.


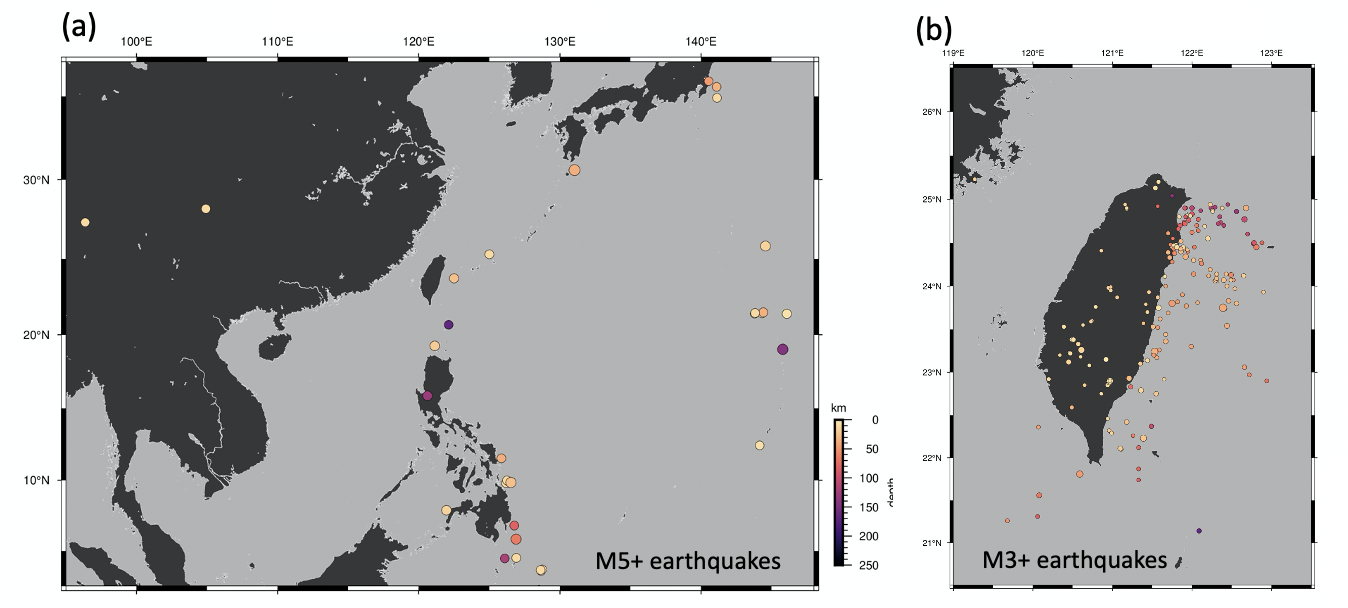


Figure S2. (a) Mapview of regional earthquakes (magnitude greater than 5) denoted by blue arrows in Figure S3. (b) Mapview of local earthquakes (magnitude greater than 3) denoted by the vertical dashed lines in Figure S3. This map is generated using GMT -5.4.4.


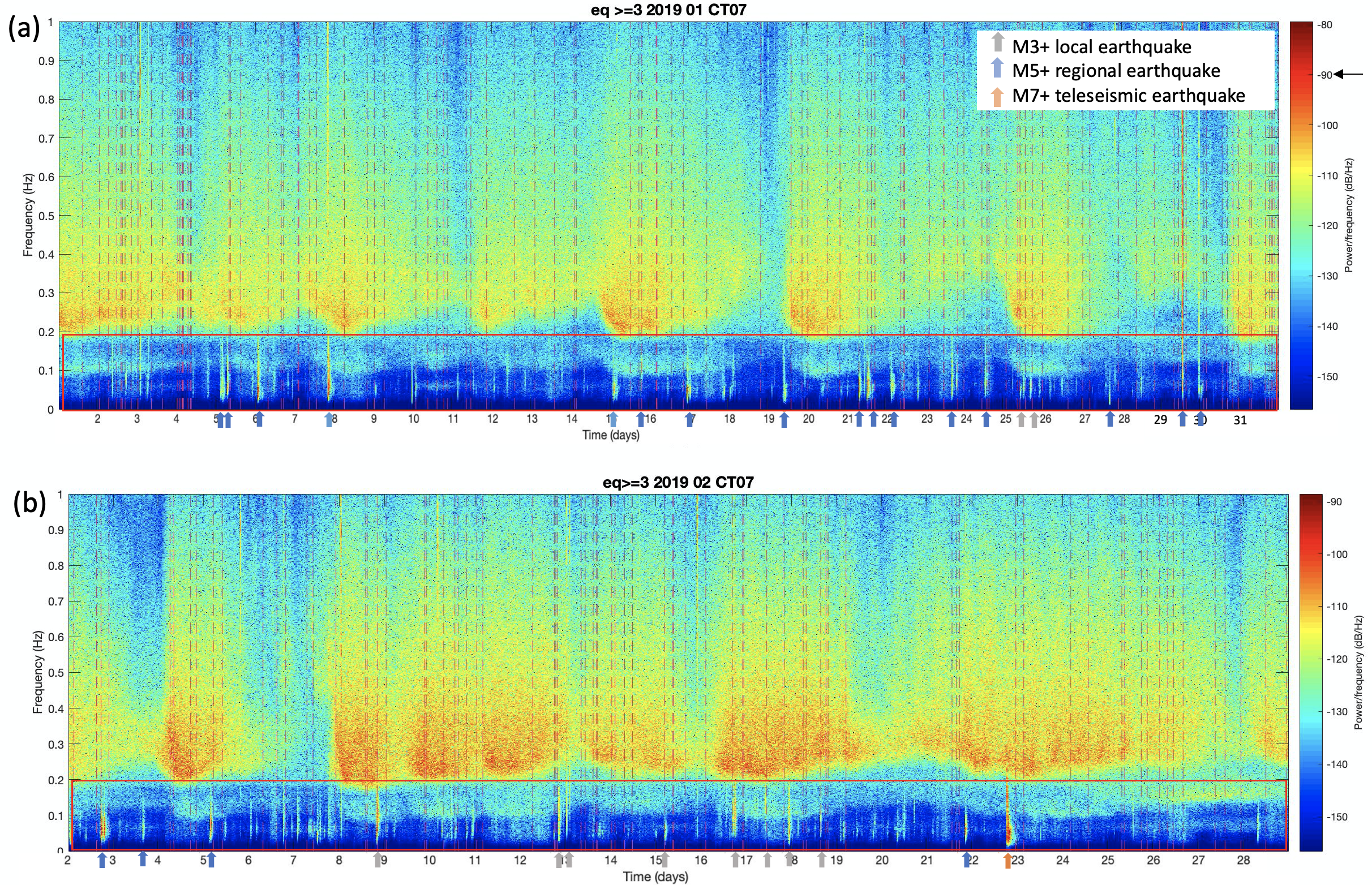


Figure S3. Spectrogram of CT07 in (a) January and (b) February. The red box denotes the frequency range of interest (below 0.2 Hz). The higher than 90 dB/Hz energy busts are denoted by arrows. Grey, blue, and orange arrows indicate a possible association with M$\geq3$local, M$\geq5$regional, and M$\geq7$ teleseismic earthquakes, respectively. The location of M$\geq3$local earthquakes and M$\geq5$regional earthquakes can be seen in Figure S2. The dashed lines indicate the occurrence time of local M$\geq3$ earthquakes (published by Central Weather Bureau earthquake catalog).


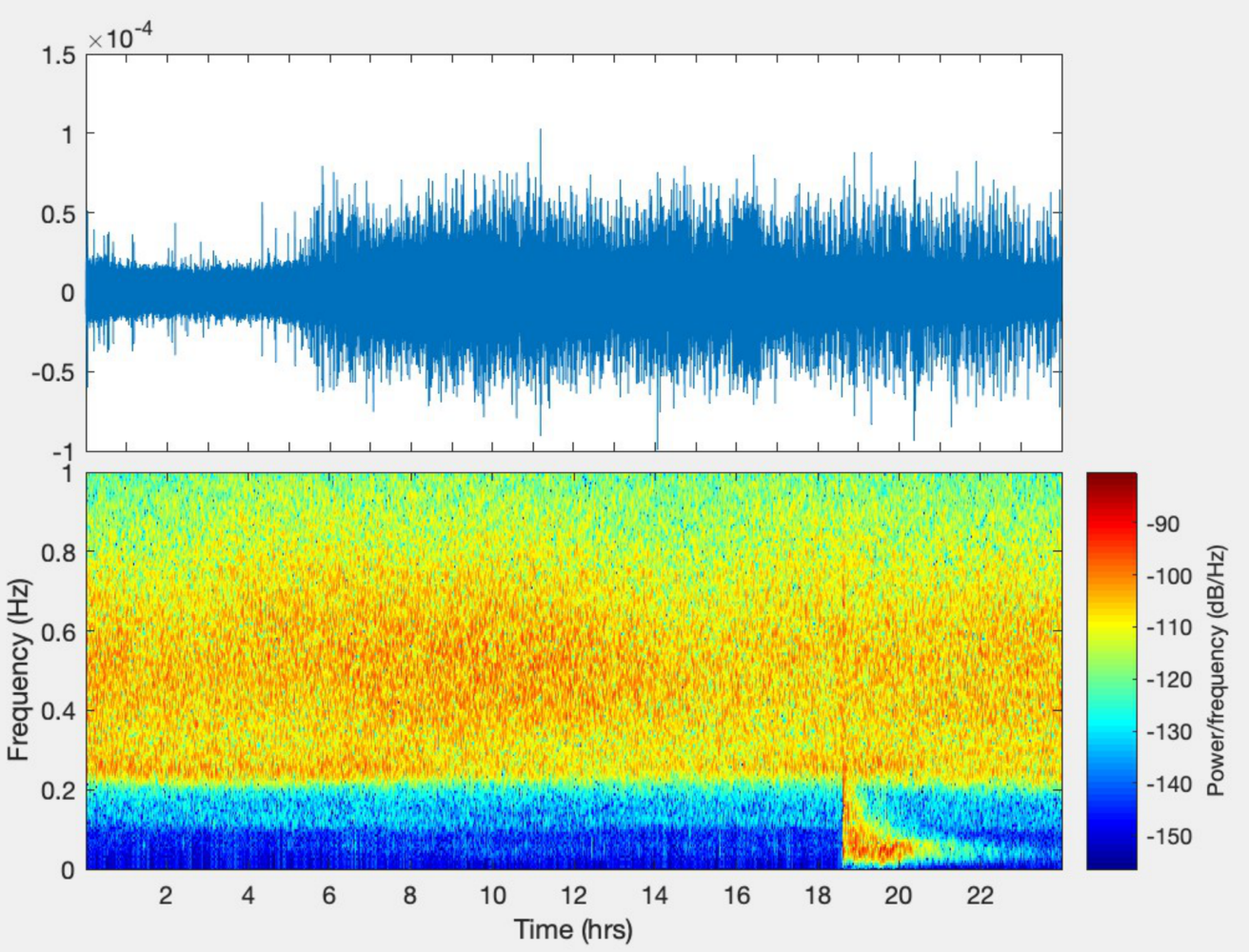


Figure S4. The waveform recorded at CT07 and the spectrogram on February 22, 2019. The low frequency, high amplitude signals can be observed to last for >1 hour without energy modulating to higher frequencies during that time. This energy burst occurred on 6:17 pm, corresponding to a M7.5 earthquake in Ecuador.


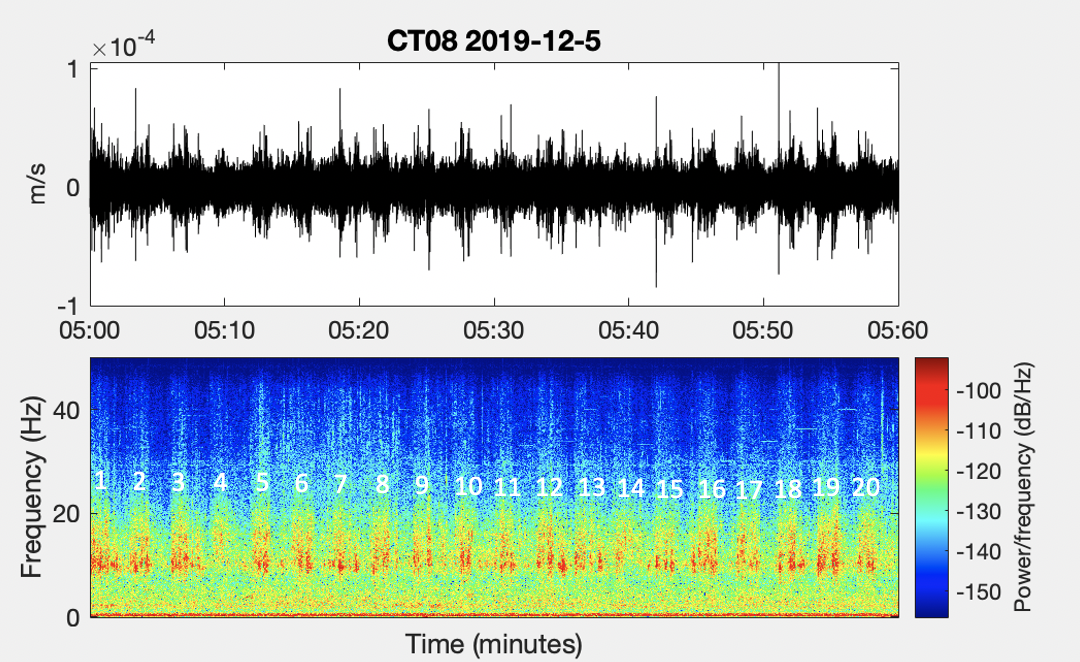


Figure S5. The original waveform recorded at CT08 and the spectrogram from 4 to 5 AM on December 5, 2019. The numbers in spectrogram denote the higher amplitude energy counted in this hour.


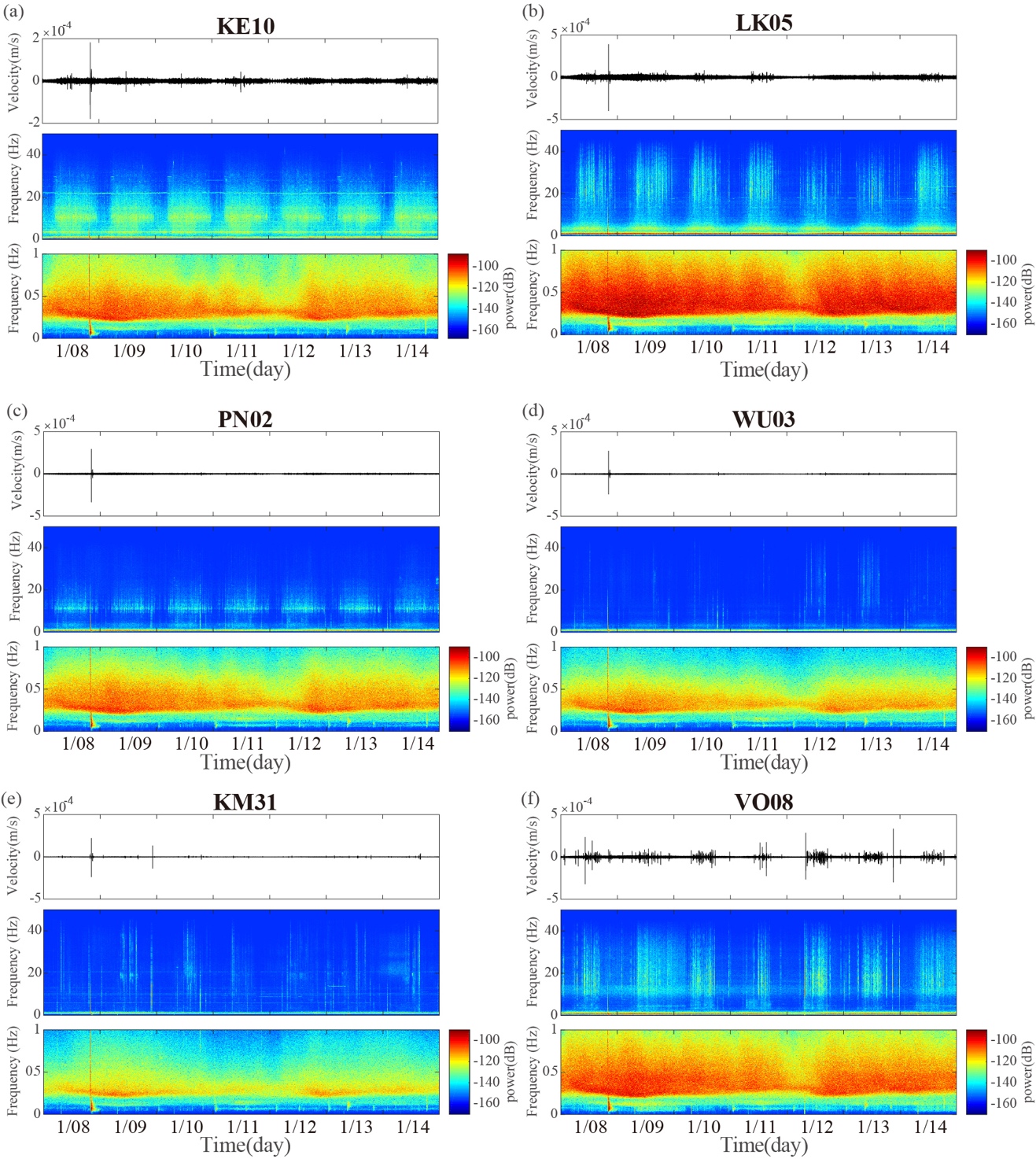


Figure S6. The spectrograms at stations located in six subareas in the surroundings of Taipei City: (a) KE, (b) LK, (c) PN, (d) WU, (e) KM, and (f) VO from January 8 to 14.


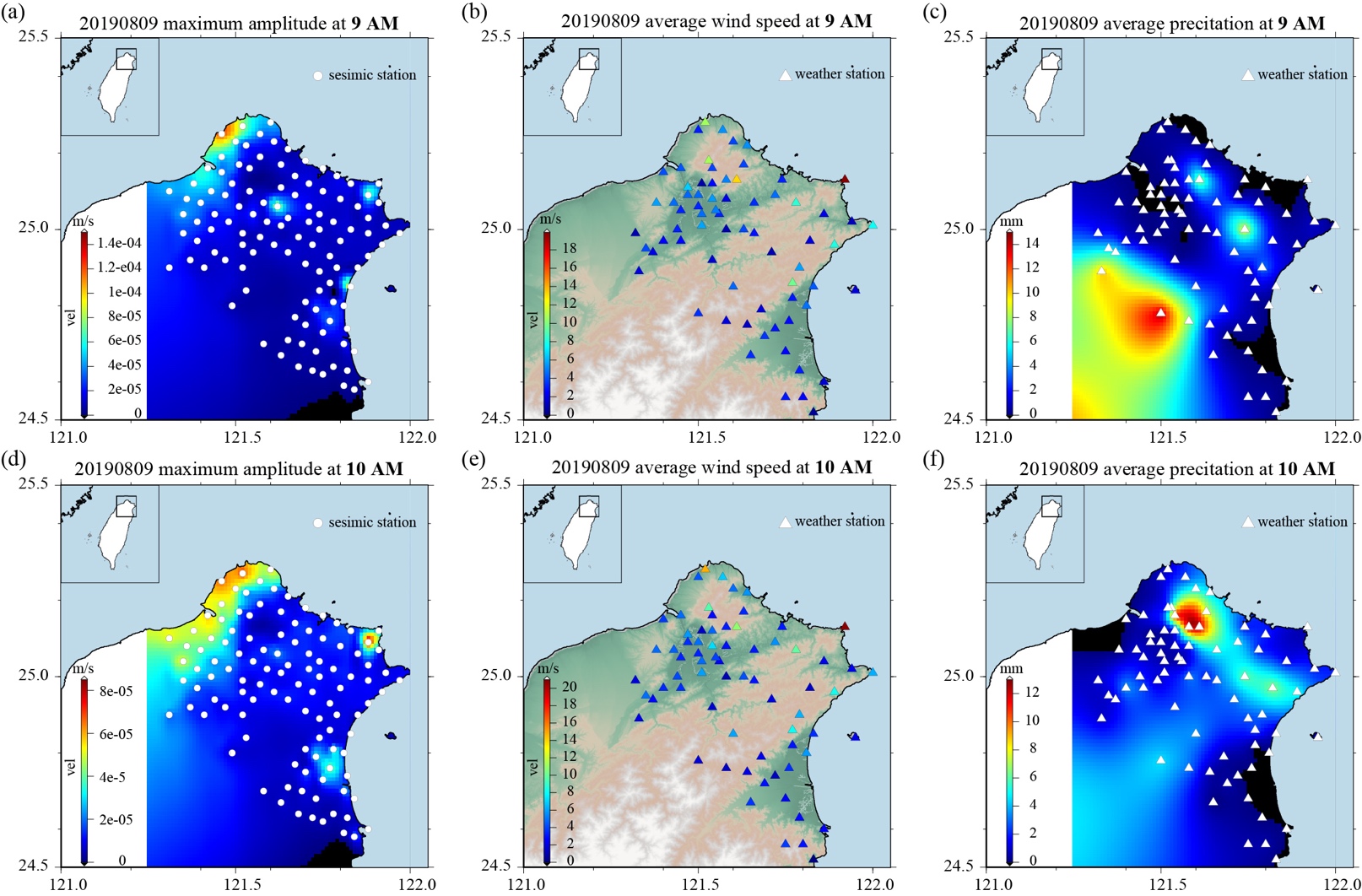


Figure S7. The distribution of (a) the maximum amplitude of 0.25-1 Hz filtered seismic data, (b) average wind velocity, and (c) average precipitation at 9 am on August 9, 2019. (d-e) Corresponding distributions at 10 am on the same date. Note that the weather stations are not evenly distributed. This map is generated using GMT -5.4.4.

**References**

Chen, W. F., and L. S. Teng (1990). Depositional environment of Quaternary deposits of the Linkou Tableland, northwestern Taiwan, *Proceedings of Geological Society of China*, 33, 39-63.

Hsiao, L. Y., Lin, K. A., Huang, S. T. and Teng, L. S. (1998). Structural characteristics of the southern Taiwan-Sinzi folded zone. *Pertroleum Geology of Taiwan*, 32,133-153. (in Chinese)

Suppe, J. (1984). Kinematics of arc-continent collision, flipping of subduction, and back-arc spreading near Taiwan, *Memoir of the Geological Society of China*, 6,21-34.

Teng, L.S., (1996). Extensional collapse of the northern Taiwan mountain belt. *Geology*, 24, 949–952.
